# Superconvergence of the local discontinuous Galerkin method for nonlinear convection-diffusion problems

**DOI:** 10.1186/s13660-017-1489-6

**Published:** 2017-09-15

**Authors:** Hui Bi, Chengeng Qian

**Affiliations:** Department of Applied Mathematics, Harbin University of Science and Technology, Harbin, 150080 China

**Keywords:** local discontinuous Galerkin method, superconvergence, convection-diffusion equations

## Abstract

In this paper, we discuss the superconvergence of the local discontinuous Galerkin methods for nonlinear convection-diffusion equations. We prove that the numerical solution is $(k+3/2)$th-order superconvergent to a particular projection of the exact solution, when the upwind flux and the alternating fluxes are used. The proof is valid for arbitrary nonuniform regular meshes and for piecewise polynomials of degree *k* ($k\geq1$). The numerical experiments reveal that the property of superconvergence actually holds true for general fluxes.

## Introduction

In this paper, we discuss the nonlinear convection-diffusion equations given by

1.1 $$ \begin{gathered} u_{t}+\partial_{x} f(u)=\nu u_{xx},\quad (x,t)\in[0,2\pi]\times[0,T], \\ u(x,0)=u_{0}(x), \quad x\in[0,2\pi], \end{gathered} $$

with the periodic boundary condition, where $\nu>0$ is a constant. We study the superconvergence of the local dicontinuous Galerkin (LDG) solutions towards a particular projection of the exact solution.

The high-order numerical methods have been applied in a variety of fields [1–4]. The LDG method is one of those numerical methods, which were first constructed by Cockburn and Shu and motivated by Bassi and Rebay [5, 6] to solve the convection-diffusion equations. Since then, the LDG method has been used to solve the time-dependent equations with high spatial derivatives, such as the Korteweg-de Vries (KdV) equations [7], time-dependent fourth-order problems [8] and the general fifth-order KdV equations [9]. See more details in [10]. We now state some theoretical results, which represent the crucial technique to treat the nonlinear parts of the equations. In [11], Zhang and Shu study the error estimate of the discontinuous Galerkin (DG) method with second-order Runge-Kutta time discretization. They obtain the optimal error estimate of the $(k+1)$th order for upwind numerical fluxes and a suboptimal error estimate of the $(k+1/2)$th order for general monotone fluxes, where *k* is the order of the piecewise polynomial space. The proof holds true for arbitrary meshes under the reasonable assumptions. Then Zhang and Shu extend the results in [11] to the third-order TVD Runge-Kutta time discretization case, which is more popular in the computation [12]. In [13], Wang and Shu obtain the optimal error estimate of the LDG method with implicit-explicit time-marching for nonlinear convection-diffusion problems, when the fluxes are chosen to be the general monotone fluxes and alternating fluxes. In the above three papers, the Taylor expansion and an *a priori* assumption are used to estimate the nonlinear parts.

We would like to mention the superconvergence results for DG and LDG methods. In [14], Cheng and Shu study the superconvergence of the $(k+3/2)$th order of the DG solution towards a particular projection for linear conservation laws. The limitations of [14] are that the proof is only valid for uniform meshes and linear piecewise polynomial space. Cheng and Shu overcome this limitation in [15], which implies that the result in [14] holds true for arbitrary meshes and *k*th-order finite element spaces. Cheng and Shu also extend the result to the linear convection-diffusion problems. For the linear equations with high-order spatial derivatives, Hufford obtains the superconvergence of the $(k+3/2)$th order for linear KdV equations [16] and Meng gets the same result for the linear fourth-order problems [17]. But for above linear problems, the numerical experiments imply that the numerical solution is superconvergent to the exact solution at a rate of the $(k+2)$th order. It is highly nontrivial to obtain this half-order increase theoretically. For linear conservation laws and linear parabolic equations, Yang and Shu use a new technique to carry out the optimal order of the superconvergence [18, 19]. In addition, they prove that DG and LDG solutions are $(k+2)$th-order superconvergent to the exact solutions at Radau points. In [20–22], Cao and Zhang present another framework to demonstrate the superconvergence at Radau points for linear 1-D and 2-D hyperbolic problems and 1-D linear parabolic problems. The first superconvergence proof with the DG method for nonlinear conservation laws is obtained in [23], when the upwind fluxes are used, under the condition that the absolute value of the convection term *f* has a positive low bound. In this paper, we obtain a similar result for the nonlinear convection-diffusion problems, when the upwind fluxes and alternating fluxes are used under the assumption that $|f'|\geq0$. Due to the character of the LDG method, there is no need of a strict positive bound of the absolute value of the convection term. In [24], Cao obtains the superconvergence of DG methods based on upwind-biased fluxes for 1-D linear hyperbolic equations. Guo and Yang show the DG solution is $(2k+1)$th-order accurate at the downwind points and $(k+2)$th-order accurate at all the other downwind-biased Radau points in [25].

The outline of this paper is as follows. In Section 2, we present the semi-discrete LDG schemes for nonlinear convection-diffusion problems. In Section 3, we state the main proofs of our theorems. Some numerical experiments are presented in Section 4, and in Section 5, we give the conclusion and our future work. Finally, we give a proof of a lemma in the Appendix.

## The local discontinuous Galerkin method for nonlinear convection-diffusion equations

### The local discontinuous Galerkin method

In this subsection, we will present the semi-discrete LDG method for equation (1.1). We first divide the computational domain $\Omega =[0,2\pi]$ into *N* subintervals. We have

$$ 0=x_{1/2}< x_{3/2}< \cdots< x_{N+1/2}=2\pi. $$

We denote each subinterval by $I_{j}=[x_{j-1/2},x_{j+1/2}]$ and the union of all $I_{j}$ by $I_{h}$. Let $h_{j}=x_{j+1/2}-x_{j-1/2}$ be the length of the subinterval and $h=\max_{1\leq j\leq N}h_{j}$. We denote the left and right limits of the function $v_{h}$ at the element interface $x_{j+1/2}$ by $(v_{h})^{-}_{j+1/2}$ and $(v_{h})^{+}_{j+1/2}$, respectively. We also set $[v_{h}]_{j+1/2}=(v_{h})^{+}_{j+1/2}-(v_{h})^{-}_{j+1/2}$ and denote the center of $I_{j}$ by $x_{j}=(x_{j+1/2}+x_{j-1/2})/2$. We would like to assume our mesh is regular, which means that there exists a constant $\lambda>0$ such that $\lambda h< h_{j}$.

We choose the finite element space as the *k*th-order piecewise polynomial space that is denoted by

$$ V^{k}_{h}= \bigl\{ v_{h}:v_{h}|_{I_{j}} \in P^{k}(I_{j}) \bigr\} , $$

where $P^{k}(I_{j})$ is the space of polynomials of degree at most *k* on $I_{j}$. In addition, we define the broken Sobolev space on $\Omega =[0,2\pi]$ as

$$ \mathcal{H}^{l}_{h}= \bigl\{ \phi:\phi|_{I_{j}}\in \mathcal{H}^{l,2}(I_{j}) \bigr\} . $$

Before we construct the LDG method, we need an auxiliary variable *q*, so we rewrite equation (1.1) as a first-order system. We have

2.1a $$\begin{aligned}& u_{t}+\partial_{x} f(u)=\sqrt{\nu}q_{x}, \end{aligned}$$

2.1b $$\begin{aligned}& q=\sqrt{\nu}u_{x}. \end{aligned}$$

Then the semi-discrete LDG scheme is formulated as follows: find $u_{h},q_{h}\in V^{k}_{h}$ such that for any $w_{h},v_{h}\in V^{k}_{h}$

2.2a $$\begin{aligned}& \begin{aligned}[b] & \int_{I_{j}}(u_{h})_{t}v_{h}\, \mathrm{d}x+\hat {f} \bigl(u^{-}_{h},u^{+}_{h} \bigr)v^{-}_{h}\big|_{j+1/2}-\hat{f} \bigl(u^{-}_{h},u^{+}_{h} \bigr)v^{+}_{h}\big|_{j-1/2}- \int_{I_{j}}f(u_{h}) (v_{h})_{x} \,\mathrm{d}x \\ &\quad=\sqrt{\nu} \biggl(\hat {q}_{h}v^{-}_{h}\big|_{j+1/2}- \hat{q}_{h}v^{+}_{h}\big|_{j-1/2}- \int_{I_{j}}q_{h}(v_{h})_{x}\, \mathrm{d}x \biggr), \end{aligned} \end{aligned}$$

2.2b $$\begin{aligned}& \int_{I_{j}}q_{h}w_{h}\,\mathrm{d}x=\sqrt{ \nu} \biggl(\hat {u}_{h}w^{-}_{h}\big|_{j+1/2}- \hat{u}_{h}w^{+}_{h}\big|_{j-1/2}- \int_{I_{j}}u_{h}(w_{h})_{x}\, \mathrm{d}x \biggr), \end{aligned}$$

where $\hat{f}(a,b)$ is usually a monotone flux, which satisfies:

- $\hat{f}(a,b)$ is consistent with the physical flux *f*, namely $\hat{f}(p,p)=f(p)$.
- $\hat{f}(a,b)$ is a Lipschitz continuous function in both arguments.
- $\hat{f}(a,b)$ is a nondecreasing function in *a* and a nonincreasing function in *b*.

Here $\hat{u_{h}}$, $\hat{q_{h}}$ are the alternating fluxes, of which we have two choices:

2.3 $$\begin{aligned}& \hat{u}_{h}=u^{-},\qquad \hat{q}_{h}=q^{+}, \end{aligned}$$

2.4 $$\begin{aligned}& \hat{u}_{h}=u^{+},\qquad \hat{q}_{h}=q^{-}. \end{aligned}$$

A new technique is necessary when the sign of the derivative of the convection term changes. Hence we only consider the case of a sign-preserving derivative, which implies that we could use the upwind flux. We have

$$ \hat{f} \bigl(p^{-}_{h},p^{+}_{h} \bigr)= \textstyle\begin{cases} f (p^{-}_{h} ),&f'(p_{h}) \geq0, \\ f (p^{+}_{h} ),&f'(p_{h})< 0. \end{cases} $$

### Norms

In this subsection, we give some norms used in this paper. We denote the standard $L^{2}$ norm on $I_{j}$ by $\|\cdot\|_{I_{j}}$. Then the norm of Sobolev space $\mathcal{H}^{l}(I_{j})$ is defined as

$$ \|u\|_{l,I_{j}}= \biggl\{ \sum_{0\leq\alpha\leq l}\big\| \mathrm{D}^{\alpha}u\big\| ^{2}_{I_{j}} \biggr\} ^{1/2}, $$

where *l* is a natural number and $\mathrm{D}^{\alpha}$ is the *α*th-order spatial derivative operator.

We denote the norm of $\mathcal{H}^{l}_{h}$ by

$$ \|u\|_{l}= \Biggl\{ \sum_{j=1}^{N} \|u\|^{2}_{l,I_{j}} \Biggr\} ^{1/2}. $$

For convenience, we set $\|\cdot\|=\|\cdot\|_{0}$. Moreover, the $L^{\infty}$ norm of the whole computational domain is

$$ \|u\|_{\infty}=\max_{1\leq j\leq N}\|u\|_{\infty,I_{j}}, $$

where $\|u\|_{\infty,I_{j}}$ is the standard $L^{\infty}$ norm on $I_{j}$. The norm on the boundary of $I_{j}$ is defined as

$$ \|u\|_{\partial I_{j}}= \bigl\{ \bigl(u^{-}_{h} \bigr)^{2}_{j+1/2}+ \bigl(u^{+}_{h} \bigr)^{2}_{j-1/2} \bigr\} ^{1/2}. $$

Then we have

$$ \|u\|_{\partial I_{h}}= \Biggl\{ \sum_{j=1}^{N} \|u\|^{2}_{\partial I_{j}} \Biggr\} ^{1/2}. $$

### Properties of the finite element space

We first present the Gauss-Radau projections, from $\mathcal {H}_{h}^{k+1}$ into $V^{k}_{h}$, which are denoted by $P^{-}_{h}$ and $P^{+}_{h}$. If $k\geq1$, we define the $P^{-}_{h}u$ to be the projection of *u* into $V^{k}_{h}$, such that for any $I_{j}$

2.5a $$\begin{aligned}& \int_{I_{j}} \bigl(u-P^{-}_{h}u \bigr)v_{h}\, \mathrm{d}x=0, \quad\forall v_{h}\in P^{k-1}(I_{j}), \end{aligned}$$

2.5b $$\begin{aligned}& P^{-}_{h}u \bigl(x^{-}_{j+1/2} \bigr)=u^{-}_{j+1/2}. \end{aligned}$$

In addition, we define $P^{+}_{h}u$ as

2.6a $$\begin{aligned}& \int_{I_{j}} \bigl(u-P^{+}_{h}u \bigr)v_{h}\, \mathrm{d}x=0,\quad\forall v_{h}\in P^{k-1}(I_{j}), \end{aligned}$$

2.6b $$\begin{aligned}& P^{+}_{h}u \bigl(x^{+}_{j-1/2} \bigr)=u^{+}_{j-1/2}, \end{aligned}$$

for any $I_{j}$.

We denote the projection error $(u-P^{-}_{h}u)$ or $(u-P^{+}_{h}u)$ by $\eta _{u}$. Thanks to the standard approximation theory [26], it is easy to obtain the following approximation property. Suppose $u(x)\in\mathcal{H}^{k+1}(I_{j})$ and is sufficiently smooth. Then we have

2.7 $$ \|\eta_{u}\|_{I_{j}}+h^{1/2}\| \eta_{u}\|_{\infty,I_{j}}+h^{1/2}\|\eta_{u}\| _{\partial I_{j}}\leq\mathcal{C}_{0}h^{k+1}_{j}, $$

where $\mathcal{C}_{0}$ is a positive constant independent of $h_{j}$.

Here and below, we use $\mathcal{C}_{0}$ to denote the corresponding constant of the estimate for projection errors. Now we turn to the three inverse properties of the finite element space $V_{h}^{k}$. For any $v_{h}\in V^{k}_{h}$, there exists a positive constant *μ* independent of *h* and *j*, such that

2.8a $$\begin{aligned}& \big\| (v_{h})_{x}\big\| _{I_{j}}\leq\mu h^{-1}\|v_{h}\|_{I_{j}}, \end{aligned}$$

2.8b $$\begin{aligned}& \|v_{h}\|_{\partial I_{j}}\leq\mu h^{-1/2} \|v_{h}\|_{I_{j}}, \end{aligned}$$

2.8c $$\begin{aligned}& \|v_{h}\|_{\infty,I_{j}}\leq\mu^{-1/2} h^{-1/2}\|v_{h}\|_{I_{j}}. \end{aligned}$$

For more details on these inverse properties, we refer the reader to [26].

### Initial projection

In order to complete the proof, an initial condition compatible with the superconvergence order would be constructed with care. Fortunately, a $(k+3/2)$th-order initial condition $\mathbb{P}_{h}$ presented in [15] is valid in our proof, that is, for any function *u*, $\mathbb{P}_{h}u\in V^{k}_{h}$. Moreover, we suppose $q_{h}\in V^{k}_{h}$ is the unique solution to

$$ \int_{I_{j}}q_{h}w_{h}\,\mathrm{d}x=( \mathbb{P}_{h}u)^{-}_{h}w^{-}_{h}\big|_{j+1/2}-( \mathbb {P}_{h}u)^{-}_{h}w^{+}_{h}\big|_{j-1/2}- \int_{I_{j}}\mathbb{P}_{h}u_{h}(w_{h})_{x} \,\mathrm{d}x, $$

for any $I_{j}$. Also, we require

2.9 $$\begin{aligned}& \int_{I_{j}} \bigl(P^{-}_{h}u-\mathbb{P}_{h}u \bigr)v_{h}\,\mathrm{d}x= \int _{I_{j}} \bigl(P^{+}_{h}q-q_{h} \bigr)v_{h}\,\mathrm{d}x,\quad\forall v_{h}\in P^{k-1}(I_{j}), \end{aligned}$$

2.10 $$\begin{aligned}& (u-\mathbb{P}_{h}u)^{-}_{j+1/2}=(q-q_{h})^{+}_{j+1/2}. \end{aligned}$$

We would like to remark that $\mathbb{P}_{h}$ exists and is unique. Moreover, we have the following estimate:

2.11 $$ \big\| P^{-}_{h}u-\mathbb{P}_{h}u\big\| \leq \mathcal{C}_{IP}h^{k+3/2}, $$

where $\mathcal{C}_{IP}=\mathcal{C}_{IP}(\|u\|_{k+2},\lambda)$ is a positive constant.

## Superconvergence

In this section, we will give the main proof of superconvergence. Similar to [13], we assume that the exact solution $u(x,t)$ is sufficiently smooth. We have

3.1 $$ \|u\|_{k+1}, \|u\|_{k+2}, \|u_{t} \|_{k+1}, \|u_{tt}\|_{k+1}\leq S, $$

where *S* is a constant independent of *t* and *h*. Also, the flux function *f* is smooth enough, for example $f\in\mathcal {C}^{3}$, and its derivatives are bounded on *R*. We have

3.2 $$ \big|f'(p)\big|, \big|f''(p)\big|,\big|f'''(p)\big| \leq\mathcal{C}_{m},\quad\forall p\in R. $$

We would like to remark that this assumption is reasonable with the original or a suitably modified flux *f*, if we only consider the smooth solution. See for more details [11].

Without loss of generality, we assume $f'>0$ and $\nu=1$. Then the fluxes are chosen as

3.3 $$ \hat{f} \bigl(u^{-}_{h},u^{+}_{h} \bigr)=f \bigl(u^{-}_{h} \bigr),\qquad \hat{u_{h}}=u^{-}_{h},\qquad \hat{q_{h}}=q^{+}_{h}. $$

For ease of notation, we denote the error between the exact solution *u* and the numerical solution $u_{h}$ by $e_{u}=u-u_{h}$. Also, we set

$$ \begin{gathered} \xi_{u}=P^{-}_{h}u-u_{h}=e_{u}- \eta_{u}, \\ \xi_{q}=P^{+}_{h}q-q_{h}=e_{q}- \eta_{q}. \end{gathered} $$

Next, we will introduce three operators, namely,

$$ \begin{gathered} \mathcal{H}^{-}_{j}(w,v)= \int_{I_{j}}wv_{x}\,\mathrm {d}x-w^{-}v^{-}\big|_{j+1/2}+w^{-}v^{+}\big|_{j-1/2}, \\ \mathcal{H}^{+}_{j}(w,v)= \int_{I_{j}}wv_{x}\,\mathrm {d}x-w^{+}v^{-}\big|_{j+1/2}+w^{+}v^{+}\big|_{j-1/2}, \\ \mathcal{K}_{j}(w,v)= \int_{I_{j}}f(w)v_{x}\,\mathrm {d}x-f \bigl(w^{-} \bigr)v^{-}\big|_{j+1/2}+f \bigl(w^{-} \bigr)v^{+}\big|_{j-1/2}.\end{gathered} $$

When the fluxes (3.3) are used, we rewrite (2.2a)-(2.2b) as

3.4a $$\begin{aligned}& \int_{I_{j}}(u_{h})_{t}v_{h}\, \mathrm{d}x-\mathcal{K}_{j}(u_{h},v_{h})=-\mathcal {H}^{+}_{j}(q_{h},v_{h}), \end{aligned}$$

3.4b $$\begin{aligned}& \int_{I_{j}}q_{h}w_{h}\,\mathrm{d}x=- \mathcal{H}^{-}_{j}(u_{h},w_{h}). \end{aligned}$$

For *u*, *q* are exact solutions, we get a similar system to (3.4a)-(3.4b), which is

3.5a $$\begin{aligned}& \int_{I_{j}}u_{t}v_{h}\,\mathrm{d}x- \mathcal{K}_{j}(u,v_{h})=-\mathcal {H}^{+}_{j}(q,v_{h}), \end{aligned}$$

3.5b $$\begin{aligned}& \int_{I_{j}}qw_{h}\,\mathrm{d}x=-\mathcal{H}^{-}_{j}(u,w_{h}). \end{aligned}$$

Then we obtain the error equations

3.6a $$\begin{aligned}& \int_{I_{j}}(e_{u})_{t}v_{h}\, \mathrm{d}x=\mathcal {K}_{j}(u,v_{h})-\mathcal{K}_{j}(u_{h},v_{h})- \mathcal{H}^{+}_{j}(e_{q},v_{h}), \end{aligned}$$

3.6b $$\begin{aligned}& \int_{I_{j}}e_{q}w_{h}\,\mathrm{d}x=- \mathcal{H}^{-}_{j}(e_{u},w_{h}). \end{aligned}$$

Due to the properties of Gauss-Radau projections and summing (3.6a)-(3.6b) over *j*, we obtain

3.7a $$\begin{aligned}& \int_{\Omega}(e_{u})_{t}v_{h}\, \mathrm{d}x=\mathcal {K}(u,v_{h})-\mathcal{K}(u_{h},v_{h})- \mathcal{H}^{+}(\xi_{q},v_{h}), \end{aligned}$$

3.7b $$\begin{aligned}& \int_{\Omega}e_{q}w_{h}\,\mathrm{d}x=- \mathcal{H}^{-}(\xi_{u},w_{h}), \end{aligned}$$

where $\mathcal{K}=\sum_{j=0}^{N}\mathcal{K}_{j}$ and $\mathcal{H}^{\pm}=\sum_{j=0}^{N}\mathcal{H}^{\pm}_{j}$.

Now we turn to an investigation of the properties of $\mathcal{H}^{\pm}(\cdot,\cdot)$ and $\mathcal{K}(\cdot,\cdot)$. By integrating by parts, we get

3.8 $$\begin{aligned}& \mathcal{H}^{-}_{j}(w,v)=- \int_{I_{j}}w_{x}v\,\mathrm {d}x-[w]_{j-1/2}v^{+}_{j-1/2}, \end{aligned}$$

3.9 $$\begin{aligned}& \mathcal{H}^{+}_{j}(w,v)=- \int_{I_{j}}w_{x}v\,\mathrm {d}x-[w]_{j+1/2}v^{-}_{j+1/2}. \end{aligned}$$

Under the periodic conditions, we have the following equation:

3.10 $$ \mathcal{H}^{-}(w,v)+\mathcal{H}^{+}(v,w)=0, $$

whose proof is straightforward.

For the nonlinear part, the derivation of the estimate is a little involving. We would like to remark that the following estimate inequality is not the final form, since we will adjust it further to obtain the suitable form in different proofs.

### Lemma 1

*Under the assumptions* (3.1) *and* (3.2), *we have the following estimate of the nonlinear part*. *For any* $v_{h}\in V^{k}_{h}$,

3.11 $$ \begin{aligned}[b] \big|\mathcal{K}(u,v_{h})- \mathcal{K}(u_{h},v_{h})\big|\leq{}&\mathcal{C}_{f}h\| \eta_{u}\|\big\| (v_{h})_{x}\big\| +\mathcal{C}_{f} \bigl(\|\xi_{q}\|+\|\xi_{u}\|+h^{-1}\|e_{u}\|_{\infty}\|e_{u}\| \bigr) \|v_{h}\| \\ &+\sum_{j=0}^{N}\bigg|f'(u_{j-1/2}) \int _{I_{j}}\eta_{q}v_{h}\,\mathrm{d}x\bigg|, \end{aligned} $$

*where* $\mathcal{C}_{f}$ *is a constant dependent on* $|f'|$, $|f''|$, *μ* *and the exact solution* *u*, *but independent of* *h*.

### Proof

We begin with using the second-order Taylor expansion with respect to the variable *u*. We have

3.12 $$\begin{aligned}& f(u)-f(u_{h})=f'(u)\eta_{u}+f'(u) \xi_{u}-\frac{1}{2}R_{f}e^{2}_{u}, \end{aligned}$$

3.13 $$\begin{aligned}& f(u)-f \bigl(u^{-}_{h} \bigr)=f'(u) \xi_{u}^{-}+f'(u)\eta^{-}_{u}-\frac{1}{2} \tilde {R}_{f} \bigl(e^{-}_{u} \bigr)^{2}, \end{aligned}$$

where

$$ \begin{gathered} R_{f}=f'' \bigl(\alpha u+(1- \alpha)u_{h} \bigr)\quad \bigl(\alpha\in(0,1) \bigr), \\ \tilde{R}_{f}=f'' \bigl(\tilde{\alpha} u+(1-\tilde{\alpha})u^{-}_{h} \bigr)\quad \bigl(\tilde {\alpha}\in(0,1) \bigr). \end{gathered} $$

We remark that we have removed the subscript $(j+1/2)$ in (3.13) for notational convenience.

Noting that $(\eta_{u}^{-})_{j+1/2}=0$, we divide $(\mathcal {K}(u,v_{h})-\mathcal{K}(u_{h},v_{h}))$ into three parts. We have

$$ \begin{aligned} \big|\mathcal{K}(u,v_{h})- \mathcal{K}(u_{h},v_{h})\big|& \leq|\Pi_{1}+ \Pi_{2}+\Pi_{3}| \\ &\leq|\Pi_{1}|+|\Pi_{2}|+|\Pi_{3}|, \end{aligned} $$

where

$$ \begin{gathered} \Pi_{1}=\sum_{j=0}^{N} \int_{I_{j}}f'(u)\eta_{u}(v_{h})_{x} \,\mathrm{d}x, \\ \Pi_{2}=\sum_{j=0}^{N} \int_{I_{j}}f'(u)\xi_{u}(v_{h})_{x} \,\mathrm{d}x-f'(u)\xi ^{-}_{u}v_{h}^{-}\big|_{j+1/2}+f'(u) \xi^{-}_{u}v^{+}_{h}\big|_{j-1/2}, \\ \Pi_{3}=-\frac{1}{2} \Biggl(\sum_{j=0}^{N} \int_{I_{j}}R_{f} e^{2}_{u}(v_{h})_{x} \, \mathrm{d}x-\tilde{R}_{f} \bigl(e_{u}^{-} \bigr)^{2}v_{h}^{-}\big|_{j+1/2}+\tilde{R}_{f} \bigl(e _{u}^{-} \bigr)^{2}v_{h}^{+}\big|_{j-1/2} \Biggr). \end{gathered} $$

We will estimate these three parts below:

- The estimate of $|\Pi_{1}|$.
  Due to property (2.5a)-(2.5b), we have
  $$ \Pi_{1}=\sum_{j=0}^{N} \int_{I_{j}} \bigl(f'(u)-f'(u_{j}) \bigr)\eta_{u}(v_{h})_{x}\,\mathrm{d}x. $$
  For $|f''|$ is bounded, it is easy to show that $|f'(u)-f'(u_{j})|\leq \mathcal{C}_{f}h$. Then we employ the Cauchy-Schwarz inequality to obtain
  3.14 $$ |\Pi_{1}|\leq\mathcal{C}_{f}h\| \eta_{u}\|\big\| (v_{h})_{x}\big\| . $$
- The estimate of $|\Pi_{2}|$.
  Proceeding as in the estimate of $|\Pi_{1}|$, we split the integration into two parts. We have
  $$ \begin{aligned} \Pi_{2}={}&\sum_{j=0}^{N} \int_{I_{j}} \bigl(f'(u)-f'(u_{j-1/2}) \bigr)\xi_{u}(v_{h})_{x}\,\mathrm {d}x \\ &- \bigl(f'(u_{j+1/2})-f'(u_{j-1/2}) \bigr)\xi_{u}^{-}v^{-}_{h}\big|_{j+1/2} \\ &+f'(u_{j-1/2}) \biggl( \int_{I_{j}}\xi_{u}(v_{h})_{x}\, \mathrm{d}x-\xi _{u}^{-}v_{h}^{-}\big|_{j+1/2}+ \xi^{-}_{u}v_{h}^{+}\big|_{j-1/2} \biggr). \end{aligned} $$
  Noting that
  $$ \int_{I_{j}}\xi_{u}(v_{h})_{x}\, \mathrm{d}x-\xi_{u}^{-}v_{h}^{-}\big|_{j+1/2}+\xi ^{-}_{u}v_{h}^{+}\big|_{j-1/2}=- \int_{I_{j}}e_{q}v_{h}\,\mathrm{d}x, $$
  with straightforward application of the Cauchy-Schwarz inequality and using properties (2.8a) and (2.8c), we obtain
  3.15 $$ \begin{aligned}[b] |\Pi_{2}|\leq{}&\sum _{j=0}^{N}\mathcal{C}h\|\xi_{u} \|_{I_{j}}\big\| (v_{h})_{x}\big\| _{I_{j}}+\big|f'(u_{j-1/2})\big| \|\xi_{q}\|_{I_{j}}\|v_{h}\|_{I_{j}} \\ &+\bigg|f'(u_{j-1/2}) \int_{I_{j}}\eta_{q}v_{h}\,\mathrm{d}x\bigg|+ \mathcal{C}h\|\xi_{u}\| _{\partial I_{j}}\|v_{h} \|_{\partial I_{j}} \\ \leq{}&\mathcal{C}_{f}\bigl(\|\xi_{u}\|+\|\xi_{q}\|\bigr) \|v_{h}\|+\sum_{j=0}^{N}\bigg|f'(u_{j-1/2}) \int_{I_{j}}\eta_{q}v_{h}\,\mathrm{d}x\bigg|. \end{aligned} $$
- The estimate of $|\Pi_{3}|$.
  Applying the Cauchy-Schwarz inequality, we obtain
  $$ \begin{aligned} |\Pi_{3}|&\leq\sum _{j=0}^{N}\mathcal{C}\|e_{u} \|_{\infty}\|e_{u}\|_{I_{j}}\big\| (v_{h})_{x} \big\| _{I_{j}}+\mathcal{C}\|e_{u}\|_{\infty}\|e_{u} \|_{\partial I_{j}}\|v_{h}\| _{\partial I_{j}} \\ &\leq\mathcal{C}\|e_{u}\|_{\infty}\|e_{u}\| \big\| (v_{h})_{x}\big\| +\mathcal{C}\|e_{u}\| _{\infty}\|e_{u}\|_{\partial\Omega}\|v_{h} \|_{\partial\Omega}. \end{aligned} $$
  In accordance with properties (2.8b) and (2.8c), we have
  3.16 $$ |\Pi_{3}|\leq\mathcal{C}_{f}h^{-1} \|e_{u}\|_{\infty}\|e_{u}\|\|v_{h}\|. $$

Collecting (3.14), (3.15) and (3.16), we obtain the estimate (3.11). □

We would like to remark that the estimate is also valid on each element $I_{j}$, though we only present the result on the whole computational domain Ω. Now we move on to the error estimate of semi-discrete LDG schemes.

### Lemma 2

*Suppose that* *u*, *q* *are the exact solutions of system* (2.1a)-(2.1b), *which are sufficiently smooth*, *and that* $u_{h}$, $q_{h}$ *are the solutions of* (2.2a)-(2.2b). *Under assumptions* (3.1) *and* (3.2), *we have the following estimate*:

3.17 $$ \frac{\mathrm{d}}{\mathrm{d}t}\|\xi_{u}\|^{2}+\| \xi_{q}\|^{2}\leq2\mathcal {C}_{f}( \mathcal{C}_{f}+3+\mathcal{C}_{e})\|\xi_{u} \|^{2}+\mathcal{C}h^{2k+2}, $$

*where* $\mathcal{C}_{e}=h^{-1}\|e_{u}\|_{\infty}+h^{-3}\|e_{u}\|^{2}_{\infty}$ *and* $\mathcal{C}$ *is a constant independent of* *h*.

### Proof

Taking $(v_{h},w_{h})=(\xi_{u},\xi_{q})$ and adding up (3.7a) and (3.7b), we have

3.18 $$ \begin{aligned}[b] \frac{1}{2}\frac{\mathrm{d}}{\mathrm{d}t}\| \xi_{u} \|^{2}+\|\xi_{q}\| ^{2}={}& \mathcal{K}(u, \xi_{u})-\mathcal{K}(u_{h}, \xi_{u})- \int_{\Omega}(\eta _{u})_{t} \xi_{u}\,\mathrm{d}x- \int_{\Omega}\eta_{q}\xi_{q}\,\mathrm{d}x \\ &-\mathcal{H}^{+}(\xi_{q},\xi_{u})-\mathcal{H}^{-}( \xi_{u},\xi_{q}). \end{aligned} $$

We deduce from property (3.10) that

$$ -\mathcal{H}^{+}(\xi_{q},\xi_{u})-\mathcal{H}^{-}( \xi_{u},\xi_{q})=0, $$

which implies that

3.19 $$ \frac{1}{2}\frac{\mathrm{d}}{\mathrm{d}t}\|\xi_{u} \|^{2}+\|\xi_{q}\|^{2}\leq \big|\mathcal{K}(u, \xi_{u})-\mathcal{K}(u_{h},\xi_{u})\big|+\bigg| \int_{\Omega}(\eta _{u})_{t} \xi_{u}\,\mathrm{d}x\bigg|+\bigg| \int_{\Omega}\eta_{q}\xi_{q}\,\mathrm{d}x\bigg|. $$

On the other hand, plugging $v_{h}=\xi_{u}$ back into (3.11), applying the Cauchy-Schwarz inequality to the integration term and using property (2.8a), we obtain

3.20 $$ \begin{aligned}[b] &\big|\mathcal{K}(u, \xi_{u})- \mathcal{K}(u_{h},\xi_{u})\big| \\ &\quad\leq\mu\mathcal{C}_{f}\| \eta_{u}\| \|\xi_{u}\|+\mathcal{C}_{f} \bigl(\| \eta_{q}\|\| \xi _{u}\|+\|\xi_{q}\|\| \xi_{u}\|+\| \xi_{u}\|^{2} \bigr) \\ &\qquad{}+\mathcal{C}_{f}h^{-1}\|e_{u} \|_{\infty}\|e_{u}\|\|\xi_{u}\|. \end{aligned} $$

Now we turn to the estimate of the last term on the right hand side of (3.20), as follows:

3.21 $$ \begin{aligned}[b] h^{-1}\|e_{u} \|_{\infty}\|e_{u}\|\|\xi_{u}\|&\leq h^{-1} \|e_{u}\|_{\infty}\bigl(\|\eta _{u}\|+\|\xi_{u} \|\bigr)\|\xi_{u}\| \\ &\leq h^{-1}\|e_{u}\|_{\infty}\|\xi_{u} \|^{2}+\mathcal{C}_{0}h^{k}\|e_{u} \|_{\infty}\| \xi_{u}\| \\ &\leq\frac{1}{4}\mathcal{C}_{0}^{2}h^{2k+3}+ \mathcal{C}_{e}\|\xi_{u}\|^{2}, \end{aligned} $$

where $\mathcal{C}_{e}=h^{-1}\|e_{u}\|_{\infty}+h^{-3}\|e_{u}\|^{2}_{\infty}$. In the first step, we use the triangle inequality and property (2.7). The proof of the last step is an application of Young’s inequality. Then we employ property (2.7) and Young’s inequality to get

3.22 $$ \mathcal{K}(u,\xi_{u})-\mathcal{K}(u_{h}, \xi_{u})\leq\mathcal{C}_{\mathcal{K}}h^{2k+2}+ \frac{1}{4}\|\xi_{q}\|^{2}+\mathcal{C}_{f}( \mathcal {C}_{f}+2+\mathcal{C}_{e})\|\xi_{u} \|^{2}, $$

where $\mathcal{C}_{\mathcal{K}}=\frac{1}{2}\mu^{2}\mathcal{C}_{f}\mathcal {C}_{0}^{2}+\frac{3}{4}\mathcal{C}_{f}\mathcal{C}^{2}_{0}$. Finally, applying property (2.7), the Cauchy-Schwarz inequality and Young’s inequality, we have

3.23 $$ \begin{aligned}[b] \bigg| \int_{\Omega}(\eta_{u})_{t}\xi_{u} \,\mathrm{d}x\bigg|+\bigg| \int_{\Omega}\eta_{q}\xi_{q}\, \mathrm{d}x\bigg|& \leq\mathcal{C}_{0}h^{k+1}\bigl(\|\xi_{u}\|+\| \xi_{q}\|\bigr) \\ &\leq\tilde{\mathcal{C}}h^{2k+2}+\mathcal{C}_{f}\| \xi_{u}\|^{2}+\frac{1}{4}\| \xi_{q} \|^{2}. \end{aligned} $$

Collecting (3.22) and (3.23), we arrive at (3.17). We thus finish our demonstration. □

To estimate $\|e_{u}\|$ and $\|e_{q}\|$, we would like to follow [23] to make the *a priori* assumption that, if *h* is sufficiently small, we have

3.24 $$ \|\xi_{u}\|\leq h^{2}. $$

It follows from property (2.8c), property (2.7) and the triangle inequality that

3.25 $$ \|e_{u}\|\leq(\mathcal{C}_{0}+1)h^{2},\qquad \|\xi_{u}\|_{\infty}\leq\mu ^{1/2}h^{3/2},\qquad \|e_{u}\|_{\infty}\leq \bigl(\mathcal{C}_{0}+ \mu^{-1/2} \bigr)h^{3/2}. $$

We will verify (3.24) at the end of this paper.

If assumption (3.24) is true, then there exists a positive constant *δ* such that

3.26 $$ \mathcal{C}_{e}=h^{-1}\|e_{u} \|_{\infty}+h^{-3}\|e_{u}\|^{2}_{\infty}\leq\delta, $$

which implies that

3.27 $$ \frac{\mathrm{d}}{\mathrm{d}t}\|\xi_{u}\|^{2}+\|\xi_{q} \|^{2}\leq\hat{\mathcal {C}}\|\xi_{u}\|^{2}+ \mathcal{C}h^{2k+2}, $$

where $\hat{\mathcal{C}}=\mathcal{C}_{f}(\mathcal{C}_{f}+3+\delta)$.

By using the Gronwall inequality and the triangle inequality, we obtain the error estimate as follows:

3.28 $$ \|\xi_{u}\|\leq\mathcal{C}h^{k+1},\qquad \|e_{u}\|\leq\mathcal{C}h^{k+1},\qquad\|e_{q}\| \leq \mathcal{C}h^{k+1}, $$

where $\mathcal{C}$ is a constant associated with $|f'|$, $|f''|$, *μ*, the exact solution *u* and the final time *T*.

In addition, we give a lemma to estimate $\|(e_{u})_{t}\|$, which will be proved in the Appendix.

### Lemma 3

*Under the assumptions* (3.1), (3.2) *and* (3.24), *we have the following estimate*:

3.29 $$ \big\| (e_{u})_{t}\big\| \leq\mathcal{C}h^{k+1}. $$

Next we will present a crucial lemma which will be used to derive the superconvergence.

### Lemma 4

*Suppose assumptions* (3.1), (3.2) *and* (3.24) *are true*. *Then we have the following inequalities*:

3.30 $$\begin{aligned}& \big\| (\xi_{u})_{x}\big\| _{I_{j}}\leq\mathcal{C}_{s} \|e_{q}\|_{I_{j}}, \end{aligned}$$

3.31 $$\begin{aligned}& \big\| (\xi_{q})_{x}\big\| _{I_{j}}\leq\mathcal{C}_{s} \bigl(\|\eta_{u}\|_{I_{j}}+\|\eta_{q}\| _{I_{j}}+\big\| (e_{u})_{t}\big\| _{I_{j}}+\| \xi_{q}\|_{I_{j}}+\|\xi_{u}\|_{I_{j}}+ \|e_{u}\|_{I_{j}} \bigr), \end{aligned}$$

*where* $\mathcal{C}_{s}$ *is a constant independent of* *h*.

### Proof

The first inequality is the same as Lemma 3.6 given in [19], so only the second inequality will be proved. Applying property (3.9) to equation (3.6a), we get

3.32 $$ \begin{aligned}[b] \int_{I_{j}}(\xi_{q})_{x}v_{h}\, \mathrm{d}x+[\xi_{q}]v_{h}^{-}\big|_{j+1/2}&= \int _{I_{j}}(e_{u})_{t}v_{h}\, \mathrm{d}x-\mathcal{K}_{j}(u,v_{h})+\mathcal {K}_{j}(u_{h},v_{h}) \\ &\leq\bigg| \int_{I_{j}}(e_{u})_{t}v_{h}\, \mathrm{d}x\bigg|+\big|\mathcal{K}_{j}(u,v_{h})-\mathcal {K}_{j}(u_{h},v_{h})\big|. \end{aligned} $$

According to properties (2.7) and (3.25), we have

3.33 $$ \big|\mathcal{K}_{j}(u,v_{h})-\mathcal{K}_{j}(u_{h},v_{h})\big| \leq\mathcal{C}\bigl(\|\eta_{u}\| _{I_{j}}+\|\eta_{q} \|_{I_{j}}+\|\xi_{q}\|_{I_{j}}+\|\xi_{u} \|_{I_{j}}+\|e_{u}\|_{I_{j}}\bigr)\| v_{h} \|_{I_{j}}. $$

Using the Cauchy-Schwarz inequality and property (2.7) yields

3.34 $$ \begin{aligned}[b] &\bigg| \int_{I_{j}}(\xi_{q})_{x}v_{h}\, \mathrm{d}x+[\xi_{q}]v_{h}^{-}\big|_{j+1/2}\bigg| \\ &\quad\leq\tilde{\mathcal{C}} \bigl(\|\eta_{u}\|_{I_{j}}+\| \eta_{q}\|_{I_{j}}+\big\| (e_{u})_{t}\big\| _{I_{j}}+\|\xi_{q}\|_{I_{j}}+\|\xi_{u} \|_{I_{j}}+\|e_{u}\|_{I_{j}} \bigr)\|v_{h} \|_{I_{j}}. \end{aligned} $$

We follow [19] to take

$$ v_{h}|_{I_{j}}=(\xi_{q})_{x}- \bigl( \xi_{q}^{-} \bigr)_{j+1/2}L \biggl(\frac{2x-2x_{j}}{h_{j}} \biggr), $$

where $L(x)$ is the *k*th-order Legendre polynomial on $[-1,1]$. Then we obtain

3.35 $$ \big\| (\xi_{q})_{x}\big\| _{I_{j}}\leq\mathcal{C}_{s} \bigl(\|\eta_{u}\|_{I_{j}}+\|\eta_{q}\| _{I_{j}}+\big\| (e_{u})_{t}\big\| _{I_{j}}+\| \xi_{q}\|_{I_{j}}+\|\xi_{u}\|_{I_{j}}+ \|e_{u}\|_{I_{j}} \bigr). $$

 □

We are now in a position to prove our theorem.

### Theorem 5

*Suppose that* *u*, *q* *are the exact solutions of* (2.1a)-(2.1b), *which are sufficiently smooth*, *and that* $u_{h}$, $q_{h}$ *are the solutions of* (2.2a)-(2.2b). *We also assume that* $f\in\mathcal{C}^{3}$ *and* $|f'|$, $|f''|$, $|f'''|$ *are bounded on* *R*. *The initial projection is chosen as* $\mathbb{P}_{h}$ *and the fluxes* (3.3) *are used in* (2.2a)-(2.2b). *For regular triangulations of* $\Omega=[0,2\pi]$, *if the piecewise polynomial space* $V^{k}_{h}$ ($k\geq1$) *is chosen to be the finite element space*, *there exists a positive constant* $h_{0}$, *such that*, *for any* $h< h_{0}$, *we have*

3.36 $$ \|\xi_{u}\|\leq\mathcal{C}_{*}h^{k+3/2}, $$

*where the positive constant* $\mathcal{C_{*}}$ *is independent of* *h*, *but maybe depends on* *u*, *f* *and* *T*.

### Proof

Recall inequality (3.19). To obtain the half-order increase, we shall use Lemma 4 to estimate the first term on the right side of (3.11). We have

3.37 $$ \mathcal{C}_{f}h\|\eta_{u}\|\cdot\big\| ( \xi_{u})_{x}\big\| \leq\mathcal{C}_{0}\mathcal {C}_{f}\mathcal{C}_{s}h^{k+2}\|e_{q}\|. $$

Set $\bar{\xi}_{u}^{j}=\frac{1}{h_{j}}\int_{I_{j}}\xi_{u}\,\mathrm{d}x$. Then, according to the orthogonality of the Gauss-Radau projection, we get

3.38 $$ \begin{aligned}[b] \sum_{j=0}^{N}\bigg|f'(u_{j-1/2}) \int_{I_{j}}\eta_{q}\xi_{u}\,\mathrm{d}x\bigg|&= \sum_{j=0}^{N}\bigg|f'(u_{j-1/2}) \int_{I_{j}}\eta_{q} \bigl(\xi_{u}-\bar{ \xi}_{u}^{j} \bigr)\,\mathrm {d}x\bigg| \\ &\leq\mathcal{C}_{m}\sum_{j=0}^{N} \|\eta_{q}\|_{I_{j}}\big\| \xi_{u}-\bar{ \xi}_{u}^{j}\big\| _{I_{j}} \\ &\leq\mathcal{C}_{m}\sum_{j=0}^{N}h \|\eta_{q}\|_{I_{j}}\big\| (\xi_{u})_{x} \big\| _{I_{j}} \\ &\leq h\mathcal{C}_{m}\mathcal{C}_{s}\sum _{j=0}^{N}\|\eta_{q}\|_{I_{j}} \|e_{q}\| _{I_{j}} \\ &\leq\mathcal{C}_{0}\mathcal{C}_{m}\mathcal{C}_{s}h^{k+2} \|e_{q}\|. \end{aligned} $$

It is a simple application of the Cauchy-Schwarz inequality to obtain the second inequality. The third one follows from the Poincaré inequality. Applying Lemma 4, the Cauchy-Schwarz inequality and property (2.7), we arrive at the last two inequalities.

Collecting (3.37), (3.38) and (3.21) and using Young’s inequality, we obtain

3.39 $$ \big|\mathcal{K}(u,\xi_{u})-\mathcal{K}(u_{h}, \xi_{u})\big|\leq\frac{1}{4}\mathcal {C}_{f} \mathcal{C}_{0}^{2}h^{2k+3}+\mathcal{C}_{q}h^{k+2} \|e_{q}\|+\frac{1}{4}\| \xi_{q}\|^{2}+ \mathcal{C}_{u}\|\xi_{u}\|^{2}, $$

where $\mathcal{C}_{q}=(\mathcal{C}_{0}\mathcal{C}_{f}\mathcal{C}_{s}+\mathcal {C}_{0}\mathcal{C}_{m}\mathcal{C}_{s})$, $\mathcal{C}_{u}=\mathcal{C}_{f}(\mathcal {C}_{f}+1+\mathcal{C}_{e})$ and $\mathcal{C}_{e}=(h^{-1}\|e_{u}\|_{\infty}+h^{-3}\|e_{u}\|_{\infty})$.

The estimates of the remaining two arguments are quite similar to (3.38), so we only present the results as follows:

3.40 $$\begin{aligned}& \int_{\Omega}(\eta_{u})_{t}\xi_{u} \,\mathrm{d}x\leq\mathcal{C}_{0}\mathcal {C}_{s}h^{k+2} \|e_{q}\|, \end{aligned}$$

3.41 $$\begin{aligned}& \begin{aligned}[b] \int_{\Omega}\eta_{q}\xi_{q}\,\mathrm{d}x\leq{}& \mathcal{C}_{0}\mathcal {C}_{s}h^{k+2} \bigl(\| \eta_{u}\|+\|\eta_{q}\|+\big\| (e_{u})_{t} \big\| +\|\xi_{u}\|+\|e_{u}\| \bigr) \\ &+\frac{1}{4}\|\xi_{q}\|^{2}+\mathcal{C}_{0}^{2} \mathcal{C}_{s}^{2}h^{2k+4}. \end{aligned} \end{aligned}$$

According to Lemma 3 and estimate (3.28), we have

3.42 $$ \frac{\mathrm{d}}{\mathrm{d}t}\|\xi_{u}\|^{2}+\|\xi_{q} \|^{2}\leq2\mathcal {C}_{f}(\mathcal{C}_{f}+1+ \mathcal{C}_{e})\|\xi_{u}\|^{2}+ \mathcal{C}h^{2k+3}. $$

Recalling estimate (3.25), we obtain

3.43 $$ \frac{\mathrm{d}}{\mathrm{d}t}\|\xi_{u}\|^{2}+\| \xi_{q}\|^{2}\leq\bar{\mathcal {C}}\|\xi_{u} \|^{2}+\mathcal{C}h^{2k+3}, $$

where $\mathcal{C}$, $\bar{\mathcal{C}}$ are constants independent of *h*.

Integrating with respect to *t*, it follows from Gronwall’s inequality and the estimate of the initial projection that

3.44 $$ \|\xi_{u}\|\leq\mathcal{C}_{*}h^{k+3/2}. $$

Finally, we will verify the *a priori* assumption (3.24) to complete our demonstration. We first mention that there exists a positive $h_{0}$, for any $h< h_{0}$, such that $\mathcal{C}_{*}h^{k+3/2}<\frac {1}{2}h^{2}$ and $\mathcal{C}_{IP}h^{k+3/2}<\frac{1}{2}h^{2}$, where $\mathcal{C}$ is the constant in (3.44) and $\mathcal{C}_{IP}$ is the constant in (2.11). Then, when $t=0$, for any $h< h_{0}$, we have

3.45 $$ \big\| \xi_{u}(\cdot,0)\big\| \leq\mathcal{C}_{IP}h^{k+3/2}< \frac{1}{2}h^{2}< h^{2}. $$

We now define

3.46 $$ M= \bigl\{ s\in[0,T]:\big\| \xi_{u}(\cdot,t)\big\| \leq h^{2},t \in[0,s] \bigr\} . $$

For *M* not empty, we denote the supremum value of *M* by $t_{\mathrm{sup}}$. If $t_{\mathrm{sup}}< T$, it follows from the continuity of $\|\xi_{u}(\cdot,t)\|$ that

3.47 $$ \big\| \xi_{u}(\cdot,t_{\mathrm{sup}})\big\| =h^{2}. $$

Then we actually have

3.48 $$ \big\| \xi_{u}(\cdot,t_{\mathrm{sup}})\big\| \leq\mathcal{C}h^{k+3/2}< \frac{1}{2}h^{2}< h^{2}, $$

which is a contradiction to (3.47). Hence, we have $t_{\mathrm{sup}}=T$, which justifies the *a priori* assumption (3.24). Thus we finish our proof. □

## Numerical experiments

In this section, we will give some numerical results to support our theorems. In all experiments, the time discretization is the third-order IM-EX Runge-Kutta scheme [13]. The time steps are chosen to be $\tau=0.5h$ in the piecewise linear polynomial case and $\tau=0.1h$ when the quadrature piecewise polynomials are used. Especially, we use more restrictive time steps, say $\tau=0.05h$, to demonstrate that smaller time steps lead to better superconvergence results, when the final time is 0.1. The initial projections are the particular projections $\mathbb{P}_{h}$ used in the proof. The following three examples have the same exact solution

4.1 $$ u(x,t)=\exp(-0.5t)\sin(x). $$

### Example 1

We first consider the following equation with periodic boundary condition:

4.2 $$ \begin{gathered} u_{t}+ \bigl(u^{3}/3 \bigr)_{x}=0.5u_{xx}+\exp(-1.5t)\sin^{2}(x) \cos(x),\quad x\in[0,2\pi], \\ u(x,0)=\sin(x). \end{gathered} $$

In this case, $f'=u^{2}>0$, which implies that we can use upwind fluxes. In Table 1 and Table 2, we present the $L^{2}$ errors of $e_{u}$ and $\xi _{u}$ and their orders on a nonuniform mesh, which is a 20% random perturbation of the uniform mesh, at the final time $T=1$ in the $P^{1}$ piecewise polynomial case and the final time $T=0.5$ in the $P^{2}$ piecewise polynomial case, respectively.

**Table 1:** **The** $\pmb{L^{2}}$ **errors and the order of the LDG method with the piecewise** $\pmb{P^{1}}$ **space at** $\pmb{T=1}$

***N***	$\boldsymbol {e_{u}}$		$\boldsymbol{\xi_{u}}$	
	$\boldsymbol{L^{2}}$ **error**	**Order**	$\boldsymbol {L^{2}}$ **error**	**Order**
10	0.0283	-	0.0030	-
20	0.0073	1.9485	3.7684e-04	2.9757
40	0.0018	2.0136	5.4173e-05	2.9889
80	4.7371e-04	1.9365	7.2521e-06	2.9011

**Table 2:** **The numerical results of the LDG method with the piecewise** $\pmb{P^{2}}$ **space at** $\pmb{T=0.1}$

***N***	$\boldsymbol {e_{u}}$		$\boldsymbol{\xi_{u}}$	
	$\boldsymbol{L^{2}}$ **error**	**Order**	$\boldsymbol {L^{2}}$ **error**	**Order**
10	0.00168	-	5.2129e-04	-
20	1.9712e-04	3.0985	3.1025e-05	4.0705
40	2.4428e-05	3.0124	2.0038e-06	3.9526

### Example 2

We take the equation

4.3 $$ \begin{gathered} u_{t}+ \bigl(0.5u^{2} \bigr)_{x}=0.5u_{xx}+0.5\exp(-t)\sin(2x),\quad x\in[-\pi,\pi], \\ u(x,0)=\sin(x), \end{gathered} $$

of which the flux function changes its sign on the computational domain. Hence, we use the Godunov flux in this example. The numerical results on the nonuniform mesh, which is a 20% random perturbation of the uniform mesh, are presented by Table 3 and Table 4, which imply that the superconvergence property is still valid in the case that the flux function is not sign preserving.

**Table 3:** **The numerical results of the LDG method with the piecewise** $\pmb{P^{1}}$ **space at the final time** $\pmb{T=1}$

***N***	$\boldsymbol {e_{u}}$		$\boldsymbol{\xi_{u}}$	
	$\boldsymbol{L^{2}}$ **error**	**Order**	$\boldsymbol {L^{2}}$ **error**	**Order**
10	0.0337	-	0.0057	-
20	0.0071	2.2469	7.9057e-04	2.8560
40	0.0018	2.0580	8.4326e-05	2.6283
80	4.6624e-04	1.9272	1.0537e-05	3.0005

**Table 4:** **The numerical results of the LDG method with the piecewise** $\pmb{P^{2}}$ **space at the final time** $\pmb{T=0.5}$

***N***	$\boldsymbol {e_{u}}$		$\boldsymbol{\xi_{u}}$	
	$\boldsymbol{L^{2}}$ **error**	**Order**	$\boldsymbol {L^{2}}$ **error**	**Order**
10	0.0018	-	6.5777e-04	-
20	1.9464e-04	2.9438	5.3149e-05	3.6295
40	2.3866e-05	3.0277	3.3170e-06	3.8606

### Example 3

In this example, we take an equation with a non-polynomial flux function. We have

4.4 $$ \begin{gathered} u_{t}+ \bigl(\exp(u) \bigr)_{x}\\ \quad=0.5u_{xx}+ \exp \bigl(\exp(-0.5t)\sin(x) \bigr) \exp(-0.5dt)\cos(x),\quad x\in[0,2\pi], \\ u(x,0)=\sin(x). \end{gathered} $$

The boundary condition is a periodic boundary condition. The mesh is also a 20% random perturbation of the uniform mesh. It results from Table 5 and Table 6 that the superconvergence property is true for a strong nonlinear flux function.

**Table 5:** **The numerical results of the LDG method with the piecewise** $\pmb{P^{1}}$ **at the final time** $\pmb{T=1}$

***N***	$\boldsymbol {e_{u}}$		$\boldsymbol{\xi_{u}}$	
	$\boldsymbol{L^{2}}$ **error**	**Order**	$\boldsymbol {L^{2}}$ **error**	**Order**
10	0.0252	-	0.0031	-
20	0.0072	1.8027	4.3012e-04	2.8375
40	0.0018	2.0197	5.2154e-05	3.2029
80	4.4483e-04	2.0049	7.0841e-06	2.8801

**Table 6:** **The numerical results of the LDG method with the piecewise** $\pmb{P^{2}}$ **at the final time** $\pmb{T=1}$

***N***	$\boldsymbol {e_{u}}$		$\boldsymbol{\xi_{u}}$	
	$\boldsymbol{L^{2}}$ **error**	**Order**	$\boldsymbol {L^{2}}$ **error**	**Order**
10	0.0016	-	4.2441e-04	-
20	1.9520e-04	3.0909	3.0008e-05	3.7303
40	2.5205e-05	2.9531	2.2068e-06	3.7653

## Conclusion

In this paper, we investigate the superconvergence of the LDG method for nonlinear convection-diffusion problems. The order of the superconvergence of the LDG method with $P^{k}$ ($k\geq1$) piecewise polynomial as the finite element space is proved to be the $(k+3/2)$th-order when the fluxes are upwind fluxes and alternating fluxes. The numerical experiments demonstrate that the superconvergence property is valid for general flux functions.

Future work includes the study of superconvergence of the LDG method for the nonlinear equations with high-order spatial derivatives in 1-D. The superconvergence properties of general monotone numerical flux will also be considered.

## Appendix

In the appendix, we will give the proof of Lemma 3, which is the estimate of $\|(e_{u})_{t}\|$.

### Proof

We begin by estimating $\|(\xi_{u})_{t}(\cdot,0)\|$. We deduce from (2.9) and (2.10) that

A.1 $$ -\mathcal{H}^{+}_{j} \bigl(\xi_{q}(\cdot,0),v_{h} \bigr)=-\mathcal{H}^{-}_{j} \bigl(\xi_{u}(\cdot ,0),v_{h} \bigr)= \int_{I_{j}}e_{q}(\cdot,0)v_{h}\, \mathrm{d}x. $$

When $t=0$, (3.7a) actually holds. If $v_{h}=(\xi_{u})_{t}$, then we have

A.2 $$ \begin{aligned}[b] \big\| (\xi_{u})_{t}(\cdot,0) \big\| ^{2}={}&{-} \int_{\Omega}(\eta_{u})_{t}( \xi_{u})_{t}\,\mathrm {d}x-\mathcal{K} \bigl(u,( \xi_{u})_{t} \bigr) \\ &+\mathcal{K} \bigl(u_{h},(\xi_{u})_{t} \bigr)+ \int_{\Omega}e_{q}(\xi_{u})_{t}\, \mathrm{d}x. \end{aligned} $$

If assumption (3.24) holds true, we employ Lemma 1, the Cauchy-Schwarz inequality and estimate (3.28) to obtain

A.3 $$ \big\| (\xi_{u})_{t}(\cdot,0)\big\| \leq\mathcal{C}h^{k+1}. $$

Differentiating (3.7a)-(3.7b) with respect of *t*, we get

A.4a $$\begin{aligned}& \int_{\Omega}(e_{u})_{tt}v_{h}\, \mathrm{d}x=\mathcal {NL}(u,u_{h},v_{h})-\mathcal{H}^{+} \bigl((\xi_{q})_{t},v_{h} \bigr), \end{aligned}$$

A.4b $$\begin{aligned}& \int_{\Omega}(e_{q})_{t}w_{h}\, \mathrm{d}x=-\mathcal{H}^{-} \bigl((\xi_{u})_{t},w_{h} \bigr), \end{aligned}$$

where

$$ \begin{aligned} \mathcal{NL}(u,u_{h},v_{h})={}& \sum_{j=0}^{N} \int_{I_{j}}\partial _{t} \bigl(f(u)-f(u_{h}) \bigr) (v_{h})_{x}\,\mathrm{d}x-\partial _{t} \bigl(f(u)-f \bigl(u^{-}_{h} \bigr) \bigr)v_{h}^{-}\big|_{j+1/2} \\ &+\partial_{t} \bigl(f(u)-f \bigl(u_{h}^{-} \bigr) \bigr)v^{+}_{h}\big|_{j-1/2}. \end{aligned} $$

Substituting $(v_{h},w_{h})=((\xi_{u})_{t},(\xi_{q})_{t})$ into (A.4a)-(A.4b) and applying property (3.10) gives

A.5 $$ \begin{aligned}[b] \frac{1}{2}\frac{\mathrm{d}}{\mathrm{d}t}\big\| ( \xi_{u})_{t}\big\| ^{2}+\big\| (\xi_{q})_{t} \big\| ^{2}\leq{}&\big|\mathcal{NL} \bigl(u,u_{h},( \xi_{u})_{t} \bigr)\big|+\bigg| \int_{\Omega}(\eta_{u})_{tt}(\xi _{u})_{t}\,\mathrm{d}x\bigg| \\ &+\bigg| \int_{\Omega}(\eta_{q})_{t}( \xi_{q})_{t}\,\mathrm{d}x\bigg|. \end{aligned} $$

We now turn to an estimate of $|\mathcal{NL}(u,u_{h},(\xi_{u})_{t})|$. The process is similar to Lemma 1, except for the fact that we use the second-order Taylor expansion, whose remainder is of the integral form, to deal with $\partial_{t}(f(u)-f(u_{h}))$. We have

$$\begin{aligned}& \begin{aligned} \partial_{t} \bigl(f(u)-f(u_{h}) \bigr)={}&\partial_{t}f'(u)\eta_{u}+f'(u) (\eta_{u})_{t}+\partial _{t}f'(u) \xi_{u} \\ &+f'(u) (\xi_{u})_{t}-\partial_{t}\mathit{IR}(e_{u})^{2}-\mathit{IR}e_{u}(e_{u})_{t} \\ ={}&\theta_{1}+\theta_{2}+\cdots+\theta_{6}, \end{aligned} \\& \begin{aligned} \partial_{t} \bigl(f(u)-f \bigl(u^{-}_{h} \bigr) \bigr)={}&\partial_{t}f'(u) \eta^{-}_{u}+f'(u) \bigl(\eta ^{-}_{u} \bigr)_{t}+\partial_{t}f'(u) \xi^{-}_{u} \\ &+f'(u) \bigl(\xi^{-}_{u} \bigr)_{t}- \partial_{t}\widetilde{\mathit{IR}} \bigl(e^{-}_{u} \bigr)^{2}- \widetilde {\mathit{IR}}e^{-}_{u} \bigl(e^{-}_{u} \bigr)_{t} \\ ={}&\sigma_{1}+\sigma_{2}+\cdots+\sigma_{6}, \end{aligned} \end{aligned}$$

where

$$ \begin{gathered} \mathit{IR}= \int_{0}^{1}(1-\beta)f'' \bigl(\beta u+(1-\beta)u_{h} \bigr) \,\mathrm{d}\beta, \\ \widetilde{\mathit{IR}}= \int_{0}^{1}(1-\tilde{\beta})f'' \bigl(\tilde{\beta} u+(1-\tilde {\beta})u^{-}_{h} \bigr) \,\mathrm{d} \tilde{\beta}.\end{gathered} $$

Then we set

$$ \begin{aligned} \big|\mathcal{NL} \bigl(u,u_{h},( \xi_{u})_{t} \bigr)\big|&=\Bigg|\sum_{i=1}^{6} \Biggl(\sum_{j=0}^{N} \int _{I_{j}}\theta_{i} \bigl((\xi_{u})_{t} \bigr)_{x}\,\mathrm{d}x-\sigma_{i}(\xi _{u})^{-}_{t}\big|_{j+1/2}+ \sigma_{i}(\xi_{u})_{t}^{+}\big|_{j-1/2} \Biggr)\Bigg| \\ &=|\Lambda_{1}+\Lambda_{2}+\Lambda_{3}+ \Lambda_{4}+\Lambda_{5}+\Lambda_{6}|. \end{aligned} $$

We are now ready to estimate each part:

- Noting the properties of the Gauss-Radau projection, we have
  A.6 $$ \sigma_{1}(x_{j+1/2})=0. $$
  Then
  A.7 $$ \begin{aligned}[b] |\Lambda_{1}|&= \Bigg|\sum _{j=0}^{N} \int_{I_{j}} \bigl(\partial_{t}f'(u)- \partial _{t}f'(u_{j}) \bigr)\eta_{u} \bigl((\xi_{u})_{t} \bigr)_{x}\,\mathrm{d}x\Bigg| \\ &\leq\mathcal{C}\|\eta_{u}\|\big\| (\xi_{u})_{t}\big\| \\ &\leq\mathcal{C}h^{2k+2}+\mathcal{C}\big\| (\xi_{u})_{t} \big\| ^{2}. \end{aligned} $$
- If we do what we did in (A.6) and (A.7), we obtain
  A.8 $$ |\Lambda_{2}|\leq\mathcal{C}h^{2k+2}+\mathcal{C}\big\| ( \xi_{u})_{t}\big\| ^{2}. $$
- Dividing the integration into two parts gives
  A.9 $$ \begin{aligned}[b] | \Lambda_{3}|={}&\Bigg|\sum_{j=0}^{N} \int_{I_{j}} \bigl(\partial_{t}f'(u)- \partial _{t}f'(u_{j-1/2}) \bigr)\xi_{u} \bigl((\xi_{u})_{t} \bigr)_{x}\,\mathrm{d}x \\ &- \bigl(\partial_{t}f'(u_{j+1/2})- \partial_{t}f'(u_{j-1/2}) \bigr) \xi^{-}_{u} \bigl(\xi _{u}^{-} \bigr)_{t}\big|_{j+1/2} \\ &+\partial_{t}f'(u_{j-1/2}) \biggl\{ \int_{I_{j}}\xi_{u} \bigl((\xi_{u})_{t} \bigr)_{x}\,\mathrm {d}x-\xi_{u}^{-} \bigl( \xi_{u}^{-} \bigr)_{t}\big|_{j+1/2}+\xi_{u}^{-} \bigl( \xi_{u}^{+} \bigr)_{t}\big|_{j-1/2} \biggr\} \Bigg| \\ \leq{}&\mathcal{C}h\|\xi_{u}\|\cdot\big\| \bigl((\xi_{u})_{t} \bigr)_{x}\big\| +\mathcal{C}h\|\xi_{u}\| _{\partial\Omega}\big\| ( \xi_{u})_{t}\big\| _{\partial\Omega}+\mathcal{C}\|e_{q} \| \cdot\big\| (\xi_{u})_{t}\big\| \\ \leq{}&\mathcal{C}h^{k+1}\big\| (\xi_{u})_{t}\big\| \\ \leq{}&\mathcal{C}h^{2k+2}+\mathcal{C}\big\| (\xi_{u})_{t} \big\| ^{2}. \end{aligned} $$
  In the second inequality, we use the Cauchy-Schwarz inequality. Using properties (2.8a) and (2.8b), we get the final step.
- Similar to the process of (A.9), we obtain
  A.10 $$ \begin{aligned}[b] |\Lambda_{4}| \leq{}&\Bigg| \sum_{j=0}^{N} \int_{I_{j}} \bigl(f'(u)-f'(u_{j-1/2}) \bigr) (\xi _{u})_{t} \bigl((\xi_{u})_{t} \bigr)_{x}\,\mathrm{d}x+f'(u_{j-1/2}) \int_{I_{j}}(e_{q})_{t}(\xi _{u})_{t}\,\mathrm{d}x \\ &+ \bigl(f'(u_{j-1/2})-f'(u_{j+1/2}) \bigr) \bigl(\xi_{u}^{-} \bigr)^{2}_{t}\big|_{j+1/2}\Bigg| \\ \leq{}&\mathcal{C}\big\| (\xi_{u})_{t}\big\| ^{2}+ \frac{1}{4}\big\| (\xi_{q})_{t}\big\| ^{2}+ \mathcal{C}h^{2k+2}. \end{aligned} $$
- If we do what we did for the estimate of $|\Pi_{3}|$, we obtain
  A.11 $$\begin{aligned}& \begin{aligned}[b] |\Lambda_{5}|&\leq\mathcal{C}h^{-1} \|e_{u}\|_{\infty}\|e_{u}\|\big\| (\xi_{u})_{t} \big\| \\ &\leq\mathcal{C}h^{k}\|e_{u}\|_{\infty}\big\| ( \xi_{u})_{t}\big\| \\ &\leq\mathcal{C}h^{2k+2}+h^{-2}\|e_{u} \|^{2}_{\infty}\big\| (\xi_{u})_{t} \big\| ^{2}, \end{aligned} \end{aligned}$$
  A.12 $$\begin{aligned}& \begin{aligned}[b] |\Lambda_{6}|&\leq\mathcal{C}h^{-1} \|e_{u}\|_{\infty}\big\| (e_{u})_{t}\big\| \big\| ( \xi_{u})_{t}\big\| \\ &\leq\mathcal{C}h^{k}\|e_{u}\|_{\infty}\big\| ( \xi_{u})_{t}\big\| +\mathcal{C}h^{-1}\|e_{u} \| _{\infty}\big\| (\xi_{u})_{t}\big\| ^{2} \\ &\leq\mathcal{C}h^{2k+2}+\mathcal{C} \bigl(h^{-1} \|e_{u}\|_{\infty}+h^{-2}\|e_{u}\| ^{2}_{\infty}\bigr)\big\| (\xi_{u})_{t} \big\| ^{2}. \end{aligned} \end{aligned}$$

Hence, we get

A.13 $$ \frac{1}{2}\frac{\mathrm{d}}{\mathrm{d}t}\big\| (\xi_{u})_{t} \big\| ^{2}+\big\| (\xi_{q})_{t}\big\| ^{2}\leq \mathcal{C}h^{2k+2}+\mathcal{C} \bigl(4+h^{-1}\|e_{u} \|_{\infty}+2h^{-2}\| e_{u}\|^{2}_{\infty}\bigr)\big\| (\xi_{u})_{t}\big\| ^{2}. $$

Following estimate (3.25), Gronwall’s inequality and the triangle inequality, we complete the proof. □

## References

[1] Wang C, Ding J, Han STW (2015). High order numerical simulation of detonation wave propagation through complex obstacles with the inverse Lax-Wendroff treatment. Commun. Comput. Phys..

[2] Wang C, Dong XZ, Shu CW (2015). Parallel adaptive mesh refinement method based on WENO finite difference scheme for the simulation of multi-dimensional detonation. J. Comput. Phys..

[3] Wang C, Zhang X, Shu CW, Ning J (2012). Robust high order discontinuous Galerkin schemes for two-dimensional gaseous detonations. J. Comput. Phys..

[4] Tan S, Wang C, Shu CW, Ning J (2012). Efficient implementation of high order inverse Lax-Wendroff boundary treatment for conservation laws. J. Comput. Phys..

[5] Cockburn B, Shu C-W (1998). The local discontinuous Galerkin method for time-dependent convection-diffusion systems. SIAM J. Numer. Anal..

[6] Bassi F, Rebay S (1997). A high-order accurate discontinuous finite element method for the numerical solution of the compressible Navier-Stokes equations. J. Comput. Phys..

[7] Yan J, Shu C-W (2002). A local discontinuous Galerkin method for KdV type equations. SIAM J. Numer. Anal..

[8] Dong B, Shu C-W (2009). Analysis of a local discontinuous Galerkin method for linear time-dependent fourth-order problems. SIAM J. Numer. Anal..

[9] Xu Y, Shu C-W (2004). Local discontinuous Galerkin methods for three classes of nonlinear wave equations. J. Comput. Math..

[10] Xu Y, Shu C-W (2010). Local discontinuous Galerkin methods for high-order time-dependent partial differential equations. Commun. Comput. Phys..

[11] Zhang Q, Shu C-W (2004). Error estimates to smooth solutions of Runge-Kutta discontinuous Galerkin methods for scalar conservation laws. SIAM J. Numer. Anal..

[12] Zhang Q, Shu C-W (2010). Stability analysis and a priori error estimates of the third order explicit Runge-Kutta discontinuous Galerkin method for scalar conservation laws. SIAM J. Numer. Anal..

[13] Wang H, Shu C-W, Zhang Q (2016). Stability analysis and error estimates of local discontinuous Galerkin methods with implicit-explicit time-marching for nonlinear convection-diffusion problems. Appl. Math. Comput..

[14] Cheng Y, Shu C-W (2008). Superconvergence and time evolution of discontinuous Galerkin finite element solutions. J. Comput. Phys..

[15] Cheng Y, Shu C-W (2010). Superconvergence of discontinuous Galerkin and local discontinuous Galerkin schemes for linear hyperbolic and convection-diffusion equations in one space dimension. SIAM J. Numer. Anal..

[16] Hufford C, Xing Y (2014). Superconvergence of the local discontinuous Galerkin method for the linearized Korteweg-de Vries equation. J. Comput. Appl. Math..

[17] Meng X, Shu C-W, Wu B (2012). Superconvergence of the local discontinuous Galerkin method for linear fourth-order time-dependent problems in one space dimension. IMA J. Numer. Anal..

[18] Yang Y, Shu C-W (2012). Analysis of optimal superconvergence of discontinuous Galerkin method for linear hyperbolic equations. SIAM J. Numer. Anal..

[19] Yang Y, Shu C (2015). Analysis of optimal superconvergence of local discontinuous Galerkin method for one-dimensional linear parabolic equations. J. Comput. Math..

[20] Cao W, Zhang Z, Zou Q (2014). Superconvergence of discontinuous Galerkin methods for linear hyperbolic equations. SIAM J. Numer. Anal..

[21] Cao W, Zhang Z (2016). Superconvergence of local discontinuous Galerkin methods for one-dimensional linear parabolic equations. Math. Comput..

[22] Cao W, Shu C-W, Yang Y, Zhang Z (2015). Superconvergence of discontinuous Galerkin methods for two-dimensional hyperbolic equations. SIAM J. Numer. Anal..

[23] Meng X, Shu C-W, Zhang Q, Wu B (2012). Superconvergence of discontinuous Galerkin methods for scalar nonlinear conservation laws in one space dimension. SIAM J. Numer. Anal..

[24] Cao W, Li D, Yang Y, Zhang Z (2017). Superconvergence of discontinuous Galerkin methods based on upwind-biased fluxes for 1D linear hyperbolic equations. ESAIM: Math. Model. Numer. Anal..

[25] Guo L, Yang Y (2017). Superconvergence of discontinuous Galerkin methods for linear hyperbolic equations with singular initial data. Int. J. Numer. Anal. Model..

[26] Ciarlet PG (1978). The Finite Element Method for Elliptic Problems.

